# A Review of Guerbet Alcohols and Their Esters: Synthesis, Applications, and Future Perspectives

**DOI:** 10.3390/ma18225180

**Published:** 2025-11-14

**Authors:** María Claudia Montiel, Salvadora Ortega-Requena, María Gómez, María Dolores Murcia, Fuensanta Máximo, Josefa Bastida

**Affiliations:** Department of Chemical Engineering, Campus de Espinardo, University of Murcia, 30100 Murcia, Spain; cmontiel@um.es (M.C.M.); dortega@um.es (S.O.-R.); maria.gomez@um.es (M.G.); md.murcia@um.es (M.D.M.); fmaximo@um.es (F.M.)

**Keywords:** Guerbet esters, branched, lubricants, cosmetics, lipase

## Abstract

Guerbet alcohol esters are compounds with specific properties that make them particularly suitable for use as cosmetic ingredients, plasticizers, or biolubricants. Guerbet alcohols are used for their synthesis. These are primary alcohols with beta branching and a lower melting point than their linear counterparts. Due to the branching, the products are liquid at lower temperatures, have good volatility, and exhibit better color and oxidation stability. This paper presents a systematic literature review on the synthesis and applications of Guerbet alcohol esters. Finally, emphasis is placed on the future of these synthesis processes, which could be based on the use of biocatalysts, thus promoting the application of new environmentally friendly procedures.

## 1. Introduction

Branched compounds offer numerous advantages over their linear analogues from both chemical and industrial perspectives. The branched structure of these molecules leads to an increase in molecular compactness, which in turn reduces the contact surface and, consequently, decreases Van der Waals forces. This results in lower boiling and melting points, which is advantageous when volatility or ease of processing is desired. Furthermore, the process of branching confers enhanced thermal and oxidative stability, as it impedes specific degradation mechanisms [1]. Among these, branched esters are notable for their extensive industrial applications which include, but are not limited to, the following: food production, cosmetics, lubricants, pharmaceuticals, biodiesel additives, and various others [2,3].

In this context, Guerbet esters represent a distinctive class of branched-chain esters that are derived from Guerbet compounds. The implementation of Guerbet-type branching within ester molecules has been demonstrated to exert a substantial influence on the extension of their liquid range, particularly at low temperatures. In light of the present availability of both Guerbet acids and alcohols, the branching point can be incorporated into the alcohol moiety, the acid moiety, or into both simultaneously [4]. However, the Guerbet esters most commonly used in industrial applications are those obtained by esterification of Guerbet alcohols with linear, branched, or dicarboxylic fatty acids [5,6]. The distinguishing characteristic of these compounds is their unique molecular structure, which typically involves a β-branched alcohol backbone. These compounds exhibit exceptional physicochemical properties, including high thermal stability, low volatility, oxidative resistance, and excellent lubricity.

This review paper presents a bibliographic search on the synthesis of Guerbet esters, highlighting their applications in different industrial fields. It also includes a collection of studies on the biocatalytic synthesis of these compounds, a growing field of research aiming to develop environmentally friendly industrial processes that comply with the 12 principles of the “Green Chemistry”.

## 2. Guerbet Alcohols

As mentioned above, Guerbet esters obtained by esterification of a Guerbet alcohol and a fatty acid are compounds of great importance because, due to their branched structure, they are widely applicable in various industrial sectors. Before summarizing the synthesis procedures and applications of these esters, it is particularly interesting to describe the origin of the branched alcohols used in them.

**Figure 1:**
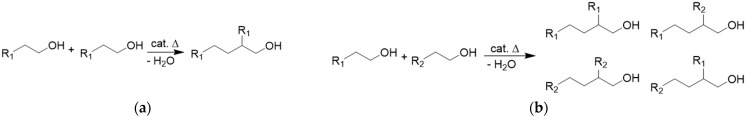
Guerbet reaction for branched alcohol synthesis. (**a**) With single alcohol; (**b**) with two different alcohols.

The Guerbet reaction, which was first described in the French journal Comptes Rendus in 1899, is named for its inventor, Marcel Guerbet [7], despite the fact that it appears to have been discovered earlier by Markovnikov [8]. The Guerbet reaction is summarily a dimerization of alcohols with liberation of water [9]. When a single alcohol is employed, the resulting reaction product is a branched alcohol that contains twice the number of carbons as the initial alcohol (see Figure 1a). In this instance, the Guerbet alcohols obtained invariably exhibit an even number of carbons, with the primary chain and the branch differing by four carbons. The reaction can also be carried out with two different alcohols, yielding a mixture of four different Guerbet alcohols as the product (Figure 1b) [10].

**Figure 2:**
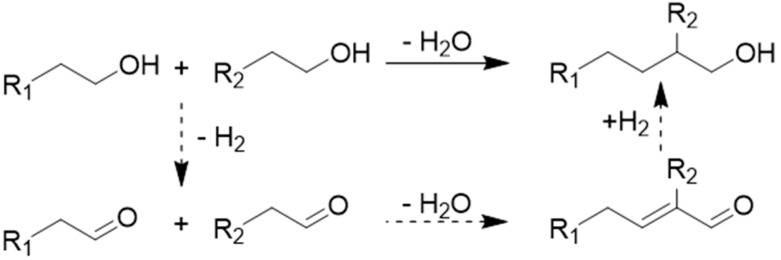
Guerbet reaction mechanism for the synthesis of branched alcohols.

The reaction mechanism is outlined in Figure 2. The sequence of steps in this process is as follows [11,12,13,14]: It begins with the dehydrogenation of a primary alcohol to form its corresponding aldehyde. This aldehyde then undergoes an aldol condensation, producing an α,β-unsaturated aldehyde intermediate. In the final stage, hydrogenation of this intermediate occurs, resulting in the formation of the desired β-branched primary alcohol, commonly known as the Guerbet alcohol [11,12,13,14]. The Guerbet reaction is thus characterized by the promotion of the first and third steps being attributable to metal catalytic sites, whereas the second step requires the involvement of acidic or basic sites.

In order to function as effective catalysts for the Guerbet reaction, such materials must be able to provide both dehydrogenation/hydrogenation functionalities and acidic/basic sites. Over the past three decades, considerable research has been dedicated to the development of catalytic systems aimed at enhancing both conversion and selectivity in the Guerbet reaction. A significant proportion of this research has focused on homogeneous and hybrid homogeneous/heterogeneous systems, with metal oxides (either as individual materials [15,16] or as mixed oxide formulations [14,17,18,19,20]). Magnesium oxide (MgO) is the most widely employed catalyst for the vapor-phase Guerbet reaction, primarily due to its strong basicity and its capability to facilitate dehydrogenation and hydrogenation processes at elevated temperatures (350–450 °C) [21,22].

The increasing availability of renewable alcohols is creating new opportunities for the advancement of Guerbet chemistry. The potential valorization of bioderived oxygenates (mainly ethanol and butanol) has led to a renewed motivation for investigating and applying the Guerbet reaction [20,21,23,24,25].

Table 1 lists the “typical” Guerbet alcohols, all of which are branched with an even number of carbons and a minimum of 6. The melting and boiling points of some of them are also summarized.

The most commonly used are C12–C36 [27]. The study of Guerbet alcohols and their applications across various domains has gained attention due to the distinct advantages they offer over their linear isomers [29]. A relevant feature of these materials is their notably lower melting point, rendering them especially well-suited for the development of functional fluids that must maintain fluidity at low temperatures. This includes specialized lubricants and hydraulic fluids utilized in aviation. Moreover, the incorporation of these compounds into jet fuel formulations has been demonstrated, further emphasizing their potential for utilization in various industrial applications [4]. In the field of cosmetics, Guerbet alcohols demonstrate notable efficacy as emollients, attributable to the branching configuration that facilitates oxygen permeability. This property is paramount for applications involving the skin. Their fully saturated molecular structure contributes to excellent oxidative and color stability, preventing rancidity and ensuring long-term performance. Furthermore, the branched configuration has been shown to reduce viscosity in comparison to linear analogues, a property that is particularly advantageous for surfactants employed in detergent formulations designed to function efficiently at low temperatures. The distinctive positioning of the branching elements within the molecule enhances its biodegradability relative to other synthetic branched alcohols, particularly when the branches consist of an even number of carbon atoms.

Current estimations indicate that the Guerbet alcohols market will attain a value of approximately USD 1.52 billion in 2025, with further growth anticipated to reach USD 2.13 billion by 2032. This expansion corresponds to a compound annual growth rate (CAGR) of 4.9% during the forecast period. At the international level, North America is expected to become the leading market, accounting for an estimated 30.3% of the global share in 2025. Within this region, the United States and Canada are the primary consumers, largely due to the significant role of the pharmaceutical and cosmetics industries, which represent the predominant end-use sectors. The development of these industries has directly contributed to the increased demand for Guerbet alcohols. In comparison, Europe is projected to represent approximately 25% of the market share by 2025. The presence of rigorous regulatory frameworks governing pharmaceutical and personal care products in this region has led to an increased demand for high-quality raw materials, including Guerbet alcohols. On the other hand, the Asia-Pacific region, comprising countries such as China, India, and Japan, is projected to account for approximately 19% of global consumption. This phenomenon is predominantly propelled by the rapid processes of industrialization, urbanization, and the expansion of the middle-class population. Specifically, China, Japan, South Korea, and India have exhibited considerable growth in the pharmaceutical and personal care sectors, thereby stimulating demand for specialty chemicals such as Guerbet alcohols. Furthermore, the escalating preeminence of the Asia-Pacific region as a nexus for chemical manufacturing has further augmented the market’s growth prospects [30].

Among the Guerbet alcohols listed in Table 1, the most commonly used in industry are: 2-butyl-1-octanol (C12), 2-hexyl-1-decanol (C16), 2-octyl-1-dodecanol (C20), 2-decyl-1-tetradecanol (C24), and 2-dodecyl-1-hexadecanol (C28), with 1-butyl-1-octanol accounting for 41.3% of the total market [30,31]. Table 2 lists the main Guerbet alcohol manufacturers and the products they offer in their portfolio.

The global market for Guerbet alcohols is marked by a high degree of consolidation and the presence of a small number of leading companies. Among these, BASF SE and Sasol Limited are particularly noteworthy, as they collectively dominate a substantial portion of the global market. Collectively, the primary competitors are responsible for over 70% of the total market share. These companies are actively expanding their production capacity, adding new facilities to meet the growing demand for this compound, especially in sectors such as cosmetics and personal care at the international level [31].

As previously indicated, Guerbet alcohols have a wide variety of applications, including their role as a starting point for other compounds of great interest, such as Guerbet acids and Guerbet esters [26]. The latter, which is the subject of this paper, will be discussed in depth in the following sections.

## 3. Guerbet Alcohol Esters

As previously mentioned, Guerbet alcohol esters are a specialized class of synthetic esters derived from Guerbet alcohols, which are branched-chain alcohols. These esters are highly valued in various industries, particularly cosmetics, lubricants and pharmaceuticals, due to their unique combination of properties.

The systematic literature review conducted in this study highlights the lack of published papers that comprehensively address the synthesis, properties, and applications of Guerbet alcohol esters. A search in the Web of Science (WOS) database using the terms “Guerbet+ester” and covering the period from 1980 to 2025 yielded a total of 137 publications. Following the exclusion of 85 patents, 52 articles remained for evaluation. Further refinement of the dataset, by discarding those not directly aligned with the search criteria, reduced the number of relevant studies to 8. These esters are outlined in Table 3, which details the Guerbet alcohol and the acid used in the esterification reaction. The table also reports the potential applications of these esters, as suggested by the respective authors, together with the corresponding bibliographic references.

Firstly, it should be noted that the few publications found do not include 2-ethyl-1-hexanol esters, despite the fact that it is a branched alcohol in the β position with a 6-carbon chain and it is strictly classified as a Guerbet alcohol. A secondary search was conducted in WOS using the terms “ethylhexanol+ester,” yielding a total of 343 results, of which 132 are patents. This indicates that the majority of researchers who have developed synthesis processes for 2-ethyl-1-hexanol esters do not consider it to be included in the Guerbet alcohol group. Therefore, in this section, only studies using alcohols with more than 12 carbon atoms and which are referred to as Guerbet in the literature have been included.

As illustrated in the table, the majority of the synthesized esters demonstrate considerable lubrication potential [5,9,10,43], exhibiting viscosity index, pour point, flash point, weld load capacity, wear scar diameter, and coefficient of friction values that are comparable to those of the most effective synthetic lubricants [5]. These properties are significantly enhanced when the esters are formed by unsaturated acids [10,41,42,43,44] or dicarboxylic acids [5,10,42]. The synthesis of hyper-branched esters using both Guerbet-type branched acids and alcohols has also been addressed [9,42], although no data on their physicochemical characteristics are provided. Therefore, their lubricating excellence cannot be confirmed. According to other authors, Guerbet alcohol esters have the potential to serve as effective metalworking fluids [45], surfactants [4], or additives for biodiesel [42].

The branched configuration of these molecules is well-known for its oxidative stability, reduced volatility, and favorable sensory characteristics, including a smooth, non-oily skin feel. Consequently, Guerbet esters are commonly incorporated into cosmetic formulations as emollients, spreading agents, and texture modifiers, enhancing the softness and absorption of creams and lotions. Their relatively high molecular weight, combined with the branching effect, ensures prolonged lubricity, making them highly suitable for advanced industrial lubricant applications. Guerbet esters are a key component in both technical and consumer-oriented products due to their unique combination of stability and multifunctionality. However, as shown in Table 3, there is a lack of publications referencing the use of Guerbet alcohol esters in the cosmetic industry. A recent search in WOS using the terms “Guerbet+ester+cosmetic” yielded 34 documents, 33 of which are patents and the remaining one refers to an award-winning research project in 2017. A subsequent manual search on the SpecialChem portal [46] established that there are 134 cosmetic ingredients on the market that include a Guerbet alcohol ester. Please refer to Table 4, which lists the esters (identified by their INCI name), the alcohols from which they are obtained, and the number of commercial cosmetic ingredients that contain the ester, either alone or in combination with other compounds.

As can be seen, most of the referenced ingredients are esters of 2-octyl-1-dodecanol (C20), which appear in 106 formulations. Among the ingredients, the myristic acid ester is the most widely used (27 ingredients), since, according to the suppliers themselves, this compound functions as an emollient and is incorporated into organic cosmetic products. It provides a non-greasy feel and low viscosity when incorporated into formulations. It is a common ingredient in various cosmetic products, including makeup, night creams, waxes, and nourishing skin care products. It is also found in many toiletries and plant-based formulations [47].

Despite the importance of Guerbet alcohol esters, as demonstrated by the studies mentioned above, there is little literature available that describes in detail the synthesis processes of these compounds. In all cases, the procedure reported is similar: place the mixture of acid and alcohol in a reactor in the presence of *p*-toluenesulfonic acid (PTSA) as a catalyst and toluene as a solvent. The mixture is heated thoroughly under vacuum or at atmospheric pressure, and conversions greater than 90% are obtained [9,10,44]. An environmental analysis of this chemical process highlights two critical points. The first is the use of an acid catalyst (PTSA), classified by the EU as an irritant to the eyes, respiratory tract, and skin [48]. In addition, it must be neutralized prior to disposal. The second point is the use of a solvent as a reaction medium. In this case, toluene is highly flammable (both the liquid and its vapors), is suspected of damaging fertility or the fetus, can be fatal if ingested and enters the respiratory tract, can cause organ damage after prolonged or repeated exposure, causes skin irritation, and can cause respiratory tract irritation or drowsiness and dizziness [48].

This context highlights the importance of developing alternative synthetic routes for the production of Guerbet alcohol esters, both to protect human health and to promote environmental sustainability. Seeking new approaches is not only motivated by the reduction in toxic reagents and extreme reaction conditions, but also by the increasing regulatory frameworks and societal demands for more responsible industrial practices. Within this scope, advances in heterogeneous catalysis, the application of green solvents, the use of renewable feedstocks, and the incorporation of biocatalytic pathways emerge as valuable strategies for cleaner and more sustainable production. At the same time, moving toward circular economy models and embracing the principles of green chemistry help mitigate the ecological footprint of these processes while enhancing industrial competitiveness in line with global market trends and the Sustainable Development Goals (SDGs) established by the United Nations. Consequently, innovation in the synthesis of Guerbet alcohol esters should be viewed not just as a technical improvement, but as a strategic necessity to secure a sustainable balance between efficiency, safety, and long-term viability.

## 4. State of the Art and Future Perspectives

Biocatalytic processes are regarded as sustainable alternatives, fully aligned with the principles of green chemistry, and are therefore seen as a key substitute for conventional production methods. Their main advantage lies in the high selectivity of the reactions and the remarkable purity of the resulting products [49]. Green chemistry provides a methodological framework that encourages continuous innovation and research in various areas of application [50]. Through the use of living organisms and enzymes, it has become possible to produce compounds on a small scale that provide significant added value, especially in the pharmaceutical industry, where regioselective synthesis is essential to achieve high levels of purity [51,52]. These techniques also open up opportunities for medium-scale industries, such as food and cosmetics, to market their products as “natural compounds” [53]. On a larger industrial scale, forecasts suggest that many plastic additives, as well as biolubricants and biodiesel, could be efficiently replaced by compounds obtained through biocatalytic synthesis [54].

Due to their broad versatility across multiple applications and their capacity for high-yield production, lipases are considered one of the most significant enzyme groups in biotechnology [55]. These enzymes exhibit remarkable substrate specificity, as well as stereoselective and enantioselective properties. This makes them widely used in biocatalytic processes [56,57]. Although lipases can be used in their natural state, their limited solubility in aqueous environments makes efficient separation from reaction media challenging. Furthermore, factors such as pH, temperature, and the intrinsic susceptibility of proteins to denaturation complicate their direct use [58]. Due to the significant impact of biocatalyst cost, strategies for enzymatic immobilization have been developed to improve activity, selectivity, and kinetic performance [59]. These techniques also facilitate catalyst recovery and ensure more effective separation of the final products.

There are few scientific publications describing the enzymatic synthesis of branched alcohol esters. In fact, searching WOS with the criteria “branched +alcohol +ester+ lipase” yields only 88 references from 1976 to 2025. However, this search criterion is ineffective because most of the articles refer to branched esters without considering whether the branching occurs in the alcohol or acid moiety. Changing the search criterion to “branched alcohol +ester+ lipase” yielded only one patent from 2008. Meanwhile, the search “Guerbet alcohol+ ester +lipase” yielded no results. In light of the findings, a search was conducted that included the names of the Guerbet alcohols utilized in the synthesis of the esters. The search “ethyl hexanol +ester +lipase OR ethyl hexyl+ ester+ lipase” referenced 86 publications since 2000. After sorting them by “Relevance,” the top 20 are listed in Table 5. Many authors do not identify 2-ethyl-1-hexanol as a Guerbet alcohol; however, these studies have been included in this paper because the results and conclusions presented have served as a foundation for subsequent work on the enzymatic synthesis of esters of branched alcohols with a higher molecular weight.

As can be seen, regardless of the origin of the lipase used, this enzyme is capable of catalyzing the synthesis of esters of 2-ethyl-1-hexanol with different saturated, unsaturated, or dicarboxylic fatty acids. It should be noted that no article describes the use of *Mucor miehei* lipase as a reaction catalyst, as it has been reported that this enzyme does not exhibit activity against 8-carbon alcohols [79]. It is also interesting to note the use of commercial immobilized lipases, among which the famous Novozym^®^ 435 [59,61,63,67,68,69,71,72,73,74,75,76] stands out, lipase B from *Candida antarctica*, which has amply demonstrated its applicability for catalyzing hydrolysis and synthesis reactions of a wide range of substrates [80,81].

Further manual searches for papers with the name of the alcohol included found only four articles describing the enzymatic synthesis of Guerbet alcohol esters with alcohols of more than eight carbons. The first study, published in 2020, details the production of 2-hexyl-1-decanol esters from a mixture of ethyl esters obtained as a fish oil byproduct, through transesterification using two methods: chemical (in a strongly basic medium) and enzymatic (using 2 commercial immobilized *Candida antarctica* B lipase, Novozym^®^ 435 and CalB immo Plus). The results obtained demonstrate the effectiveness of enzymatic synthesis in the case of large alcohols when the reaction is carried out at 60 °C and 10 mbar. Furthermore, the reaction proceeds with equal effectiveness (conversion >90% after 6 h) whether the reaction is carried out in the presence or absence of solvents. Finally, the authors highlight the advantage of reusing the enzyme which results in a reduction in process costs [82].

The second publication details the optimization of the production process of myristic, palmitic, and stearic esters of 2-octyl-1-dodecanol *with Candida antarctica* B lipase, Lipozyme^®^ 435 [83]. The authors indicate that these three esters are used in the cosmetics industry as substitutes for cyclomethcones since, in recent years, it has been shown that some of them (those with lower molecular weight) have recently been classified as persistent, bioaccumulative, and toxic (PBT) substances [84]. In this case, the authors chose to carry out the process in a solvent-free reaction medium, at atmospheric pressure and temperatures ranging from 70–90 °C. The results obtained show that conversions of over 95% are achieved in reaction times of between 3–4 h. In addition, the easy separation by sedimentation of the immobilized enzyme and the purity of the product obtained (>98%) facilitate the separation and purification processes. In addition, the sustainability of the process is highlighted by the calculated “green metrics” values, emphasizing the low value of the Environmental Factor (<0.04), which demonstrates the minimal waste generation caused by the biocatalytic process [83].

The remaining two articles detail the biocatalytic synthesis of the hyperbranched ester 2-octyl-1-dodecanoyl-2-methylhexanoate [85,86]. Enzymatic synthesis was carried out in an open air reactor and at 80 °C, achieving 95% conversion after 5 h of reaction. The authors propose this compound as an excellent biolubricant and highlight the significant advantage of branches in improving the lubricating properties of these esters. This superiority is quantitatively demonstrated by the compound’s notably high Viscosity Index (VI) of 204, which exceeds the values reported in the literature for many synthetic and mineral lubricants. The viscosity index (VI) is an essential measure of lubricant performance, as higher values (around 200) indicate that the lubricant maintains a relatively stable viscosity across a wide temperature range. By comparison, conventional mineral oils usually have VIs between 90 and 100, while standard biolubricants extend this range to approximately 140–150 [87,88]. Some high-performance vegetable oils, such as soybean oil (VI 236), industrial-grade oleic acid (VI 208) [86], and especially castor oil (*Ricinus communis*, VI 320) [89], show even higher VIs. However, the main advantage of this ester lies in the fact that it is a saturated molecule, which gives it greater stability against oxidation.

These two recent studies [85,86] have revealed new possibilities for producing other saturated esters with high viscosity indexes. It has been shown that enzymatic catalysis can be used in the absence of solvents to esterify a branched acid, such as 2-methylhexanoic acid, with other branched alcohols. This process is particularly relevant because the biocatalytic reaction offers high selectivity, avoids the use of solvents, and achieves high conversion rates, making it a sustainable, environmentally friendly alternative.

## 5. Conclusions

In conclusion, the present review highlights the considerable potential of Guerbet alcohol esters as multifunctional compounds with distinct physicochemical properties, particularly low volatility and enhanced stability, which render them suitable for applications in cosmetics, lubricants, and pharmaceuticals. These advantages are largely attributable to their branched-chain structure, which distinguishes them from conventional esters. Recent progress in biocatalytic and enzymatic methodologies provides promising avenues for the sustainable synthesis of these compounds, aligning with the principles of Green Chemistry and emphasizing the importance of environmentally benign production strategies. Nevertheless, the current body of literature remains limited with respect to the systematic study of their synthesis, properties, and applications, underscoring a critical gap in knowledge. Addressing these deficiencies through targeted research could facilitate the optimization of synthetic pathways, the identification of novel industrial uses, and the assessment of long-term impacts. Collectively, the findings presented herein advocate for increased industrial attention to Guerbet alcohol esters, with the aim of advancing both scientific understanding and sustainable technological applications.

## Figures and Tables

**Table 1:** Guerbet alcohols (adapted from [26,27,28]).

Guerbet Alcohol	Formula	Melting Point (°C)	Boiling Point (°C) (p, kPa)	Reference
2-Methyl-1-pentanol	C_6_H_14_O		147.9 (101.3)	[27]
2-Ethyl-1-hexanol	C_8_H_18_O	<−76/−70	118 (10.7)	[26,27]
2-Propyl-1-heptanol	C_10_H_22_O		117 (2.7)	[27]
2-Butyl-1-octanol	C_12_H_26_O	≈−30	126–128 (1.5)	[27,28]
2-Pentyl-1-nonanol	C_14_H_30_O	≈−25	154 (1.7)	[27,28]
2-Hexyl-1-decanol	C_16_H_34_O	−30 to −26	175 (1.5)	[27]
2-Heptyl-1-undecanol	C_18_H_38_O	−26	198 (2)	[27]
2-Octyl-1-dodecanol	C_20_H_42_O	−20	135–137 (0.007)	[27]
2-Nonyl-1-tridecanol	C_22_H_46_O	≈10	164–167 (0.013)	[27,28]
2-Decyl-1-tetradecanol	C_24_H_50_O	≈18	173–175 (0.007)	[27,28]
2-Undecyl-1-pentadecanol	C_26_H_54_O			
2-Dodecyl-1-hexadecanol	C_28_H_58_O	32–39	203–207 (0.007)	[27,28]
2-Tridecyl-1-heptadecanol	C_30_H_62_O			
2-Tetradecyl-1-octadecanol	C_32_H_66_O	38–39	308–310 (2.0)	[26,27,28]
2-Pentadecyl-1-nonadecanol	C_34_H_70_O			
2-Hexadecyl-1-eicosanol	C_36_H_74_O	43–45	270–280 (0.013)	[26,27]
2-Heptadecyl-1-heneicosanol	C_38_H_78_O			
2-Octadecyl-1-docosanol	C_40_H_82_O			
2-Nonadecyl-1-tricosanol	C_42_H_86_O			
2-Eicosyl-1-tetracosanol	C_44_H_90_O			

**Table 2:** Main suppliers of Guerbet alcohols and products offered in their portfolio.

Key Companies in the Market	Trade Name	Reference
BASF SE	Lutensol^®^ XL (40, 50, 70, 79, 80, 90, 100) Lutensol^®^ XP (30, 40, 50, 70, 79, 80, 89, 90) (ethers based on C10 Guerbet alcohol)	[32]
Sasol Ltd.	ISOFOL 12 (2-butyl-1-octanol) ISOFOL 16 (2-hexyl-1-decanol) ISOFOL 18T (2-octyl-1-decanol) ISOFOL 18E (2-octyl-1-decanol) ISOFOL 20 (2-octyl-1-dodecanol) ISOFOL 24 (2-decyl-1-tetradecanol) ISOFOL 2426S (C24 and C26) ISOFOL 28 (2-dodecyl-1-hexadecanol) ISOFOL 32 (2-tetradecyl-1-octadecanol)	[33]
New Japan Chemical Co., Ltd.	NJCOL 160BR (2-hexyl-1-decanol) NJCOL 200A (2-octyl-1-dodecanol) NJCOL 240A (2-decyl-1-tetradecanol)	[34]
Kao Corporation	FINDET LI/1990 FINDET LR4/2585 (Polyoxyethylene fatty branched alcohol)	[35]
Kisco Ltd.	2-Butyl-1-octanol 2-Octyl-1-dodecanol	[36]
Kokyu Alcohol Kogyo Co., Ltd.	RISONOL 24SP (2-decyl-1-tetradecanol)	[37]
DowPol Corporation	Guerbet Alcohol G 16 (2-hexyl-1-decanol) Guerbet Alcohol G 20 (2-octyl-1-dodecanol)	[38]
Aurorium (formerly Vertellus. The company acquired Jarchem Industries in 2021)	Jarcol ™ I-12 (2-butyl-1-octadecanol) Jarcol ™ I-16CG (2-hexyl-1-decanol) Jarcol ™ I-20H (2-octyl-1-dodecanol) Jarcol ™ I-20P (2-octyl-1-dodecanol) Jarcol ™ I-20N (2-octyl-1-dodecanol) Jarcol ™ I-24 (2-decyl-1-teradecanol) Jarcol ™ I-28CG (2-dodecyl-1-hexadecanol) Jarcol ™ I-28 (2-dodecyl-1-hexadecanol)	[39]
Emco Dyestuff P. Ltd.	XL (140, 100, 90, 80, 70, 60, 50) (ethers based on C10 Guerbet alcohol)	[40]

**Table 3:** Synthesis and applications of Guerbet alcohol esters.

Guerbet Alcohol	Acid	Application	Reference
2-Butyl-1-octanol (C12)	Waste cooking oil	Biolubricant for drilling fluids	[41]
	C18 Guerbet acid C24 Guerbet acid	Lubricant	[9]
	Glutaric acid 2,2-Diglutaric acid Adipic acid Suberic acid Palmitic acid Stearic acid Oleic acid	Biodiesel additive	[42]
	Epoxidized soybean oil	Lubricant	[43]
	Coco-oleic estolide	Lubricant	[44]
2-Pentyl-1-nonanol (C14)	C18 Guerbet acid	Lubricant	[9]
	Epoxidized soybean oil	Lubricant	[43]
2-Hexyl-1-decanol (C16)	C18 Guerbet acid	Lubricant	[9]
	Adipic acid Sebacic acid	Lubricant	[5]
	Glutaric acid 2,2-Diglutaric acid Adipic acid Suberic acid Sebacic acid Palmitic acid Stearic acid Oleic acid	Biodiesel additive	[42]
	Epoxidized soybean oil	Lubricant	[43]
	Coco-oleic estolide	Lubricant	[44]
2-Heptyl-1-decanol (C17)	Oleic acid Erucic acid Adipic acid Sebacic acid	Lubricant	[10]
2-Heptyl-1-undecanol (C18)	C12 Guerbet acid C16 Guerbet acid C18 Guerbet acid	Lubricant	[9]
	Oleic acid Erucic acid Adipic acid Sebacic acid	Lubricant	[10]
	Epoxidized soybean oil	Lubricant	[43]
2-Octyl-1-dodecanol (C20)	C18 Guerbet acid C24 Guerbet acid	Lubricant	[9]
	Adipic acid Sebacic acid	Lubricant	[5]
	Eicosanoic acid C20 Guerbet acid	Surfactant	[4]
	Coco-oleic estolide	Lubricant	[44]
2-Decyl-1-tetradecanol (C24)	C18 Guerbet acid	Lubricant	[9]
	Butyric acid Lauric acid Stearic acid	Metalworking fluids	[44]
	Adipic acid Sebacic acid	Lubricant	[5]

**Table 4:** Cosmetic ingredients containing Guerbet alcohol esters.

Alcohol	Guerbet Alcohol Ester	Number of Cosmetic Ingredients
2-Butyl-1-octanol (C12)	Butyloctyl salicylate	17
2-Hexyl-1-decanol (C16)	Hexyldecyl laurate	4
	Hexyldecyl stearate	1
	Hexyldecyl ethylhexanoate	3
	Hexyldecyl isostearate	2
2-Octyl-1-dodecanol (C20)	Octyldodecyl stearoyl stearate	14
	Octyldodecyl myristate	27
	Octyldodecyl neopentanoate	5
	Octyldodecyl PCA	4
	Octyldodecyl stearate	4
	Octyldodecyl xyloside	3
	Octyldodecyl oleate	5
	Octyldodecyl citrate	1
	Octyldodecyl lactate	4
	Octyldodecyl olivate	1
	Octyldodecyl ricinoleate	3
	Octyldodecyl isostearate	5
	Octyldodecyl lanolate	2
	Octyldodecyl/lauroyl glutamate	11
	Octyldodecyl hydroxystearate	1
	Octyldodecyl erucate	3
	Octyldodecyl neodecanoate	1
	Octyldodecyl benzoate	1
	Octyldodecyl behenate	1
2-Decyl-1-tetradecanol (C24)	Decyltetradecyl myristoyl methyl beta-alaninate	1

**Table 5:** Ethylhexyl esters obtained by enzymatic synthesis.

Ester	Lipase	Application	Reference
Di-2-ethylhexyl adipate	Novozym^®^ 435 Lipozyme^®^ IM	Synthetic lubricant	[60]
2-Ethylhexyl palmitate	*Candida* sp. 99-125 lipase	Cosmetics, pharmaceutics, and food and chemical industries	[61]
Di-2-ethylhexyl sebacate	Novozym^®^ 435 Lipozyme^®^ IM Porcine pancreas lipase	-	[62]
2-Ethylhexyl palmitate	*Candida* sp. 99-125 lipase	Cosmetic, pharmaceutic, food and chemical industries	[63]
2-Ethylhexyl palmitate	Novozym^®^ 435	Lubricant and plasticizer	[64]
2-Ethyl-1-hexyl palmitate	*Candida antarctica* (CAL A)	-	[65]
2-Ethylhexyl esters of fatty acids of rapeseed oil	*Candida antarctica* lipase *Pseudomonas cepacia* lipase *Rhizomucor miehei* lipase	Lubricant	[66]
2-Ethylhexyl palmitate	*Candida* sp. 99-125 lipase	Cosmetic, pharmaceutical, food, and chemical industries	[67]
2-Ethylhexyl ferulate	Novozym^®^ 435	Antioxidant in pharmaceutical, cosmetic and food industries	[68]
Diethylhexyl adipate	Novozym^®^ 435	Paint stripper, fragrance, perfume, lubricant, food packaging, and plasticizer	[69]
2-Ethylhexyl oleate	Novozym^®^ 435	Green plasticizer	[70]
2-Ethylhexyl stearate	Fermase CALB 10000	Cosmetic biolubricant	[71]
2-Ethylhexyl oleate	Novozym^®^ 435	Emollient	[72]
Bis(2-ethylhexyl) azelate	Novozym^®^ 435	Lubricant	[73]
Ethylhexyl ester of waste cooking oil	Novozym^®^ 435	Lubricant	[74]
2-Ethylhexyl palmitate 2-Ethylhexyl stearate	Novozym^®^ 435 Novozym^®^ 40086	Natural alternatives of cyclomethicone in cosmetics	[75]
Ethylhexyl ester of γ-linolenic acid	Novozym^®^ 435	Cosmetic ingredient	[76]
2-Ethylhexyl 2-methylhexanoate	Novozym^®^ 435	Cosmetic ingredient	[77]
2-Ethylhexyl oleate	*Candida antarctica* lipase	Pharmaceutical, food, cosmetics, and chemical industries	[78]
2-Ethylhexyl esters of castor oil fatty acids	Eversa Transform 2.0 lipase *Candida antarctica* CAL A *Candida antarctica* CAL B *Rhizomucor miehei* lipase *Thermomyces lanuginosus* lipase	Biolubricant	[79]

## Data Availability

No new data was created or analyzed in this study. Data sharing is not applicable to this article.

## Abbreviations

The following abbreviations are used in this manuscript:

CAGR	Compound Annual Growth rate
EU	European Union
INCI	International Nomenclature of Cosmetic Ingredients
PBT	Persistent, bioaccumulative, and toxic
PTSA	*p*-Toluenesulfonic acid
SDGs	Sustainable Development Goals
USD	United States Dollar
VI	Viscosity Index
WOS	Web of Science
